# Amyloid Peptide *β*1-42 Induces Integrin *α*IIb*β*3 Activation, Platelet Adhesion, and Thrombus Formation in a NADPH Oxidase-Dependent Manner

**DOI:** 10.1155/2019/1050476

**Published:** 2019-03-14

**Authors:** Aisha Alsheikh Abubaker, Dina Vara, Caterina Visconte, Ian Eggleston, Mauro Torti, Ilaria Canobbio, Giordano Pula

**Affiliations:** ^1^Pharmacy and Pharmacology, University of Bath, UK; ^2^Institute of Biomedical and Clinical Sciences, University of Exeter Medical School, UK; ^3^Department of Biology and Biotechnology, University of Pavia, Italy

## Abstract

The progression of Alzheimer's dementia is associated with neurovasculature impairment, which includes inflammation, microthromboses, and reduced cerebral blood flow. Here, we investigate the effects of *β* amyloid peptides on the function of platelets, the cells driving haemostasis. Amyloid peptide *β*1-42 (A*β*1-42), A*β*1-40, and A*β*25-35 were tested in static adhesion experiments, and it was found that platelets preferentially adhere to A*β*1-42 compared to other A*β* peptides. In addition, significant platelet spreading was observed over A*β*1-42, while A*β*1-40, A*β*25-35, and the scA*β*1-42 control did not seem to induce any platelet spreading, which suggested that only A*β*1-42 activates platelet signalling in our experimental conditions. A*β*1-42 also induced significant platelet adhesion and thrombus formation in whole blood under venous flow condition, while other A*β* peptides did not. The molecular mechanism of A*β*1-42 was investigated by flow cytometry, which revealed that this peptide induces a significant activation of integrin *α*IIb*β*3, but does not induce platelet degranulation (as measured by P-selectin membrane translocation). Finally, A*β*1-42 treatment of human platelets led to detectable levels of protein kinase C (PKC) activation and tyrosine phosphorylation, which are hallmarks of platelet signalling. Interestingly, the NADPH oxidase (NOX) inhibitor VAS2870 completely abolished A*β*1-42-dependent platelet adhesion in static conditions, thrombus formation in physiological flow conditions, integrin *α*IIb*β*3 activation, and tyrosine- and PKC-dependent platelet signalling. In summary, this study highlights the importance of NOXs in the activation of platelets in response to amyloid peptide *β*1-42. The molecular mechanisms described in this manuscript may play an important role in the neurovascular impairment observed in Alzheimer's patients.

## 1. Introduction

Alzheimer's disease (AD) is a multifactorial age-related neurodegenerative disorder representing 60-80% of dementia cases [1]. Prominent morphological hallmarks of the disease include pathological accumulation of insoluble aggregates of polymeric protein fragments known as *β* amyloid peptides deposited in the brain parenchyma (amyloid plaques) and within the cerebral vessel walls (cerebral amyloid angiopathy (CAA)), formation of neurofibrillary tangles within neurons (tau pathology), oxidative stress and chronic neurovascular inflammation resulting in blood hypoperfusion, and damages to the blood brain barrier (BBB) [2]. The manifestation of these pathological conditions eventually lead to neurovascular dysfunction, neuronecrosis, cognitive decline, and ultimately death [3].

Epidemiological data, postmortem pathological examination, and experimental studies on both human and animal AD brains have revealed significant correlations and shared pathophysiological mechanisms between Alzheimer's and vascular diseases [4–9]. Common contributing causes include conditions such as hypertension, diabetes mellitus, hypercholesterolemia, apolipoprotein E (APOE) 4 polymorphism, and traumatic brain injury [10].

The potential role of platelets in Alzheimer's disease has been investigated in a number of studies. The initial work of Rosenberg et al. in 1997 highlighted possible platelet activation in AD patients due to altered APP processing [11]. His work was followed up by Sevush et al. in 1998 and by other groups later on, and it was confirmed that there is an aberrant and chronic preactivation of platelets that can eventually contribute towards atherothrombosis, CAA, and progression of AD [12]. Several studies showed a correlation between AD and platelet abnormalities, including abnormal membrane fluidity, increased *β*-secretase activity, and altered APP metabolism [13]; *α*-degranulation, P-selectin surface expression, and integrin *α*IIb*β*3 activation [14]; platelet adhesion [15, 16]; formation of leukocyte-platelet complexes [12]; coagulation abnormalities [17–19]; and platelet adhesion and accumulation at vascular *β* amyloid deposition sites, where they were shown to modulate *β* amyloid complexation into aggregates [20].

Several authors utilised both soluble and fibril forms of *β* amyloid peptides as agonists and demonstrated that A*β* peptides are able to promote platelet activation, adhesion, and aggregation. For example, fibrillar A*β*1-40 was shown to induce platelet aggregation by binding to scavenger receptors CD36 and GP1b*α* and activating p38 MAPK/COX1 pathways. This induces the release of the potent aggregation agonist thromboxane A2 (TxA2) [21]. Donner et al. more recently showed that A*β*1-40 can bind to integrin *α*IIb*β*3 and trigger the release of ADP and clusterin (a chaperone protein), which promoted the formation of A*β*1-40 fibrils [22]. In addition, the use of synthetic A*β*25-35, which retains the biological and toxic properties of the full length A*β*1-40 and A*β*1-42, has been shown to activate the PAR1 thrombin receptor and stimulate an intracellular signalling cascade involving Ras/Raf, PI3K, P38MAPK, and cPLA2 and TxA2 formation and release [23].

NADPH oxidases (NOXs) are the only enzyme family recognized for their sole primary function of generating reactive oxygen species (ROS), and they have been proposed as the main source of ROS in platelets during haemostasis [24]. Recently, two types of NOXs have been identified in human and mouse platelets (NOX1 and NOX2) [25], but a comprehensive understanding of their activation signalling pathways in response to *β* amyloid peptides remains elusive. An interesting paper published by Walsh et al. demonstrated that oligomeric and fibrillar forms of A*β*1-42 can act as a ligand for the GPVI receptor and activate platelets [26]. Since NOX1 has been shown to play a key role in signalling for the GPVI receptor [25, 27], this may suggest that A*β*1-42 acts through a NOX1-dependent activation of platelets.

Recently, we demonstrated that upon stimulation of platelets by both monomeric or fibril forms of A*β*1-42, significant intracellular superoxide anion formation can be detected using a novel flow cytometry method using the molecular probe dihydroethidium (DHE) [28]. Here, we further investigate the effects of different A*β* (A*β*1-42, A*β*1-40, A*β*25-35, and scrambled A*β*1-42 control) on platelet activation and adhesion in static and physiological flow conditions. The use of pan-NOX inhibitor VAS2870 [28] allows the assessment of the role of NOXs on platelet adhesion and activation. Our primary objective is to understand the mechanism underlining *β* amyloid peptide-dependent regulation of platelets, which can potentially improve our understanding of AD and facilitate the development of pharmacological tools to combat the progression of this disease.

## 2. Materials and Methods

### 2.1. Reagents

Dimethylsulfoxide (DMSO), indomethacin, prostaglandin E_1_ (PGE_1_), bovine serum albumin (BSA), sodium citrate solution (4% w/v), fibrinogen, thrombin from human plasma, 4% w/v paraformaldehyde, TRITC-conjugated phalloidin, 3,3′-dihexyloxacarbocyanine iodide (DiOC6), VAS2870, D-(+)-glucose monohydrate, and 4-(2-hydroxyethyl) piperazine-1-ethanesulfonic acid (HEPES) were from Sigma-Aldrich (Poole, UK). Fibrillar collagen was from Chrono-Log Corporation (Havertown, PA US). The anti-phosphotyrosine antibody (4G10) was from Upstate Biotechnology Inc. (Lake Placid, US). Anti-PKC phosphor-substrate antibody was from Cell Signaling Technology (Danvers, US). Anti-pleckstrin antibody was from Abcam (Cambridge, UK). FITC-PAC1 and PE-Cy5-CD62P (P-selectin) antibodies were from Becton Dickinson, (Wokingham, UK). Peroxidase-conjugated anti-IgG antibodies were from Bio-Rad (Hercules, US). The chemiluminescent substrate kit was from Merck Millipore (Burlington, US). Amyloid peptides were synthesized by LifeTein (New Jersey, US). The sequences of the peptides are as follows:

- A*β*1-40 (4.3 kDa): DAEFRHDSGYEVHHQKLVFFAEDVGSNKGAIIGLMVGGVV
- A*β*1-42 (4.5 kDa): DAEFRHDSGYEVHHQKLVFFAEDVGSNKGAII GLMVGGVVIA
- Scrambled A*β*1-42: (4.5 kDa): DEFAKNIGHHDGVAVHMYKGRQVEFIGSIALVFEDVGSAGLV
- A*β*23-35 (1.0 kDa): GSNKGAIIGLM

### 2.2. Preparation of Washed Platelets

Human whole blood was obtained from healthy volunteers following Royal Devon and Exeter NHS Foundation Trust Code of Ethics and Research Conduct and under NRES South West – Central Bristol committee approval (Rec n. 14/SW/1089). 20–30 ml of blood were drawn in the presence of the anticoagulant sodium citrate (0.5% w/v). Platelet rich plasma (PRP) was then isolated from whole blood by centrifugation at 200 × g for 20 mins. PRP was then subjected to a second centrifugation at 500 × g for 10 mins in the presence of indomethacin (10 *μ*M) and PGE_1_ (40 ng/ml). Platelets were resuspended in modified Tyrode's HEPES buffer (145 mM NaCl, 2.9 mM KCl, 10 mM HEPES, 1 mM MgCl_2_, and pH 7.3; 5 mM D-glucose was added before use).

### 2.3. Adhesion Assay

Round coverslips (22 mm) were placed in a clear flat-bottom 6-well plate and then coated overnight at 4°C with 10 *μ*M A*β*1-42, A*β*1-40, A*β*25-35, scrambled A*β*1-42, or 5 mg/ml BSA, all diluted in PBS. Excess solution was then gently removed, and the coverslips were blocked with 0.5% w/v BSA in PBS for 1 h at room temperature. Coated dishes were then washed gently with PBS. Washed platelets were resuspended at a density of 2 × 10^7^ platelets/ml. 0.5 ml of washed platelets were incubated for 30 mins at 37°C. Nonadherent platelets were discarded, and the adherent ones were gently washed with PBS and then fixed with 4% w/v paraformaldehyde for 10 mins at RT. 0.1% v/v Triton X-100/PBS was added to permeabilize the platelets. After 5 mins, 0.1% Triton X-100/PBS was removed, and the coated coverslips were washed with PBS and then blocked with 5 mg/ml BSA in PBS for 30 mins. Fixed platelets were then stained with 10 *μ*M TRITC-conjugated phalloidin for 1 hour at RT and then washed with PBS. Coverslips were mounted onto microscope slides using Vectashield. Evaluation of platelet adhesion and spreading was performed using a Leica LED fluorescence microscope, and digital images were acquired at 10x and 100x magnification objectives. Platelet coverage and surface area were measured using ImageJ (version 1.52e, Wayne Rasband, NIH).

### 2.4. Thrombus Formation Assay under Physiological Flow

Human peripheral blood was anticoagulated with sodium citrate 0.25% w/v. Platelets were fluorescently labelled by incubation with 1 *μ*M 3,3′-dihexyloxacarbocyanine iodide (DiOC6) for 10 minutes. ibidi Vena8 Fluoro+ microchips (ibidi GmbH, Martinsried, Germany) were coated with 10 *μ*M A*β* peptides or 0.1 mg/ml fibrillar collagen. Nonspecific binding sites were saturated with 0.1% w/v BSA. Physiological flow conditions (200–1000 sec^−1^) were applied using an ExiGo pump (Cellix Ltd. Microfluidics Solutions, Dublin, Ireland). Images of the thrombi formed after 10 minutes of flow were obtained with an EVOS Fl microscope (Thermo Fisher Scientific, Waltham, MA, US). Platelet coverage was measured using ImageJ (version 1.52e, Wayne Rasband, NIH).

### 2.5. Flow Cytometry

Platelets isolated as described above were resuspended at 2 × 10^7^ cells/ml density. After stimulation in suspension as described (5–20 *μ*M A*β*1-42, A*β*1-40, A*β*25-35, scrambled A*β*1-42, or 0.5 unit/ml thrombin) for 10 minutes at 37°C, platelets were incubated for a further 10 minutes with PAC1 and anti-P-selectin antibodies conjugated to FITC and PE-Cy5, respectively. 1 in 10 dilution in Tyrode's buffer was used to stop the immunolabelling of the platelets. Surface fluorescence was assessed using a FACSAria III flow cytometer (BD Biosciences, San Jose, USA).

### 2.6. Immunoblotting

Platelet samples (0.2 ml, 1 × 10^9^ platelets/ml) were stimulated at 37°C under stirring conditions (1,200 rpm) with A*β*1-42, A*β*1-40, and A*β*25-35 (including 1 mM CaCl_2_) in a 490D aggregometer (Chrono-Log Corporation, Havertown, PA, US). Where indicated, the NOX inhibitor VAS2870 (10 *μ*M) was preincubated for 10 minutes before treatments with A*β* peptides. The reaction was stopped after 3 minutes by the addition of a half volume of 3x SDS sample buffer (37.5 mM Tris, pH 8.3, 288 mM glycine, 6% SDS, 1.5% DTT, 30% glycerol, and 0.03% bromophenol blue) followed by heating the samples at 95°C for 5 minutes. Platelet proteins were separated on SDS-PAGE gels, transferred to a PVDF membrane, and analysed in immunoblotting using anti-phosphotyrosine antibody (4G10), anti-phospho-PKC substrate antibody, and anti-pleckstrin antibodies. Reactive proteins were visualized by ECL.

### 2.7. Statistical Analysis

Data were analysed by one-way ANOVA with Bonferroni posttest using the statistical software GraphPad Prism. Results were expressed as the mean ± standard error (SEM). Differences were considered significant at *P* value < 0.05.

## 3. Results

**Figure 1:**
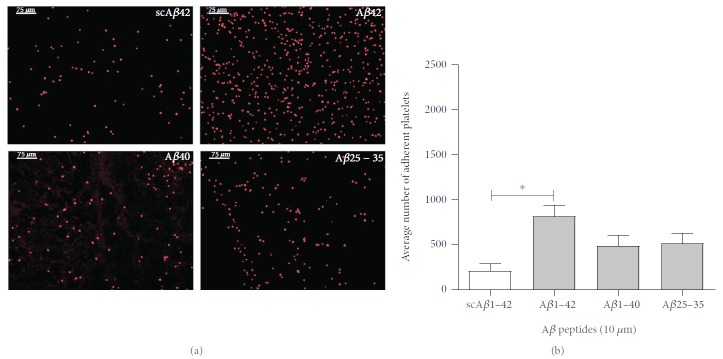
*β* amyloid peptides in static conditions support platelet adhesion. Human platelet suspensions were plated on glass coverslips coated with 10 *μ*M scrambled A*β*1-42 (scA*β*42), A*β*1-42, A*β*1-40, and A*β*25-35. The adhered platelets after 30 minutes shown in (a) were fixed, permeabilized, and stained with TRITC-conjugated phalloidin and are representative images at 10x magnification. The quantification of the adhered platelets evaluated as the mean number of platelets per optical field is shown in (b). Statistical significance for 4 independent experiments was analysed by one-way ANOVA with Bonferroni posttest (^∗^*P* < 0.05), with bars representing standard error of the mean (SEM).

Initial experiments were carried out to examine the effect of amyloidogenic peptides on human platelet adhesion in static conditions by allowing platelet suspensions to rest on coverslips coated with scrambled A*β*1-42, A*β*1-42, A*β*1-40, and A*β*25-35 for 30 minutes. Adhering platelets were then fixed, permeabilized, and stained with TRITC-phalloidin. Phalloidin is a poisonous molecule extracted from the mushroom *Amanita phalloides* that binds strongly to filaments of actin (F-actin), which is a major component of the cell cytoskeleton [29]. In these experiments, phalloidin allows effective visualization of adhered platelets.  Figure 1(a) shows the high number of adhered platelets to A*β*1-42. Adhesion to A*β*1-40, or A*β*25-35 is higher than control scrambled A*β*1-42 peptide, but this difference is not statistically significant. The quantification of adhered platelets from 4 independent experiments is shown in Figure 1(b). The statistical analysis (one-way ANOVA with Bonferroni posttest) shows significance (*P* < 0.05) for the increase in platelet adhesion to A*β*1-42 compared to scrambled A*β*1-42.

**Figure 2:**
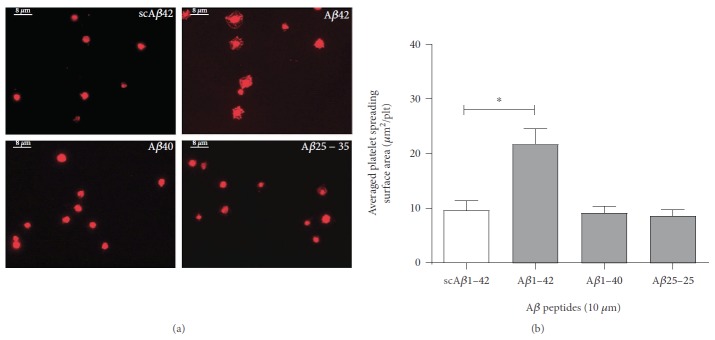
*β* amyloid peptides in static conditions support platelet spreading. Human platelet suspensions were plated on glass coverslips coated with 10 *μ*M scrambled A*β*1-42 (scA*β*42), A*β*1-42, A*β*1-40, and A*β*25-35. The adhered platelets shown in (a) were fixed, permeabilized, and stained with TRITC-conjugated phalloidin and are representative images at 100x magnification. The quantification of the mean surface area of the adhered platelets (*μ*m^2^/plt) is shown in (b). Statistical significance for 4 independent experiments was analysed by one-way ANOVA with Bonferroni posttest (^∗^*P* < 0.05), with bars representing standard error of the mean (SEM).

In order to assess platelet spreading on A*β* peptides, Figure 2(a) displays the morphological changes in adhering platelets at a higher magnification (100x). Initial adhesion is characterised by shape change from discoid into irregular or round shape and filopodia formation, while complete adhesion and signalling activation is associated with extensive platelet spreading and formation of lamellipodia. A*β*1-40 and A*β*25-35 induced only the morphological changes of the early phase of platelet adhesion, with few fine processes extending into different directions (filopodia). On the other hand, platelets adhering to A*β*1-42 show full spreading, with the formation of extensive lamellipodia and a significant increase in surface area coverage.  Figure 2(b) quantifies the average surface area of the platelets confirming the effectiveness of A*β*1-42 as a substrate for signalling activation and platelet spreading, while no platelet spreading is observed for A*β*1-40 and A*β*25-35.

**Figure 3:**
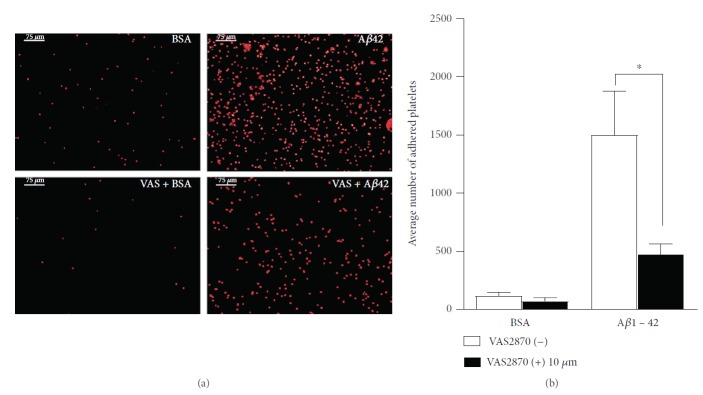
Effect of NOX inhibitor VAS2870 on platelet adhesion to A*β*1-42. Human platelet suspension was preincubated with NOX inhibitor VAS2870 (10 *μ*M) for 30 mins then plated on glass coverslips coated with 10 *μ*M A*β*1-42 and 5 mg/ml BSA in PBS. The numbers of the adhered platelets fixed, permeabilized, and stained with TRITC-conjugated phalloidin are shown in (a), and representative images at 10x magnification are displayed. The quantification of the adhered platelets evaluated as the mean number of the adhered platelets per optical field is shown (b). Statistical significance for 4 independent experiments was analysed by one-way ANOVA with Bonferroni posttest (^∗^*P* < 0.05), with bars representing standard error of the mean (SEM).

Since platelets demonstrated significant adhesion and spreading on A*β*1-42, we next investigated the effects of the NOX inhibitor VAS2870 on this substrate. Both platelet adhesion and spreading were strongly impaired. In Figure 3(a), we show the quantification of platelets adhering to A*β*1-42 in the presence or absence of VAS2870. Although the number of adhering platelets is not completely abated by VAS2870, there was a statistically significant decrease in the number of adhering platelets in the presence of this inhibitor (Figure 3(b)).

**Figure 4:**
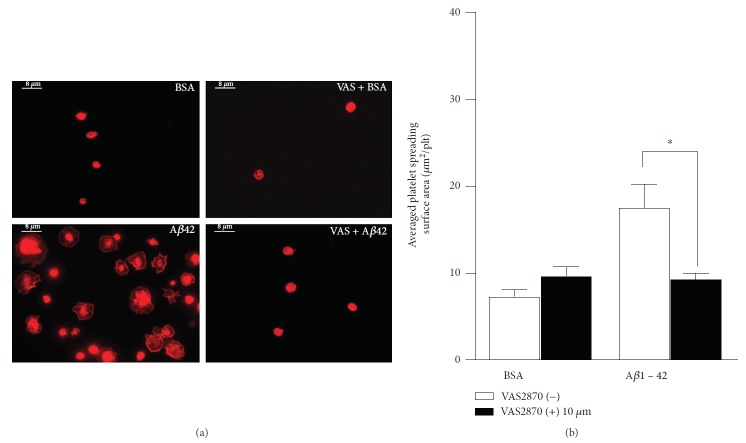
Effect of NOX inhibitor VAS2870 on platelet spreading on A*β*1-42. Human platelet suspension was preincubated with NOX inhibitor VAS2870 (10 *μ*M) for 30 mins then plated on glass coverslips coated with 10 *μ*M A*β*1-42 and 5 mg/ml BSA in PBS. The surface area of the adhered platelets fixed, permeabilized, and stained with TRITC-conjugated phalloidin is shown in (a), and representative images at 100x magnification are displayed. The quantification of the surface area of the adhered platelets (*μ*m^2^/plt) is shown in (b). Statistical significance for 4 independent experiments was analysed by one-way ANOVA with Bonferroni posttest (^∗^*P* < 0.05), with bars representing standard error of the mean (SEM).

When observing adhering platelets at a higher magnification (100x), VAS2870 appeared to significantly reduce the platelet spreading and the resulting surface area per platelet was lower than in the absence of the NOX inhibitor (Figure 4(a)). Statistical analysis revealed statistical significance of the effect of VAS2870 on platelet spreading (Figure 4(b)).

**Figure 5:**
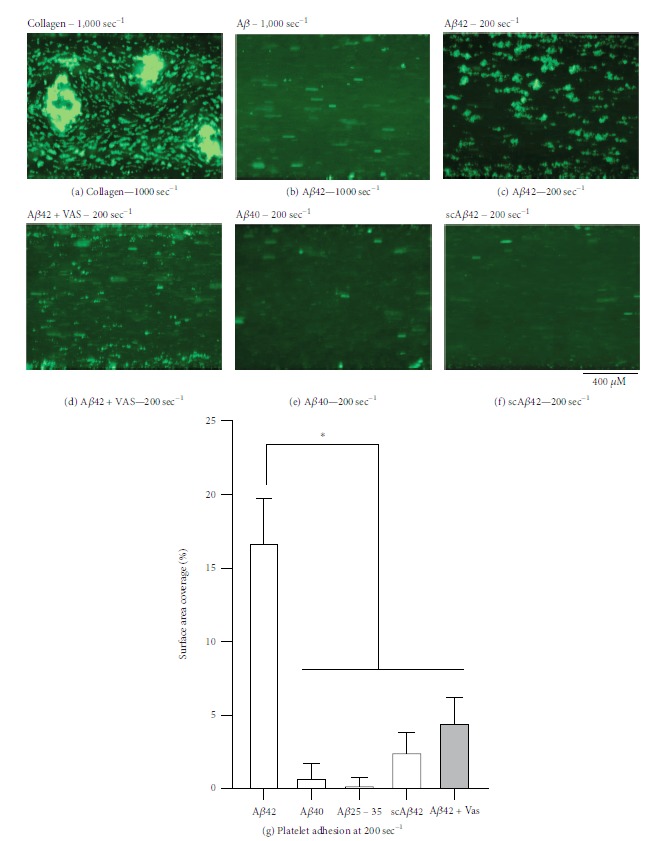
Adhesion of platelets to amyloid peptides under physiological shear stress. Flow biochips (ibidi Vena8 Fluoro+) were coated with 0.1 mg/ml fibrillary collagen or 10 *μ*M scrambled A*β*1-42 (scA*β*42), A*β*1-42, A*β*1-40, and A*β*25-35. Platelet adhesion was tested in human whole blood at shear rates of 1,000 sec^−1^ and 200 sec^−1^. Where indicated, 10 *μ*M VAS2870 was added to the blood to inhibit NOXs. Pictures shown here are representative of 3 independent experiments. Surface coverage analysis was performed using ImageJ and statistically analysed by one-way ANOVA with Bonferroni posttest (^∗^*P* < 0.05).

Next, we tested whether A*β* peptides can stimulate platelet adhesion under physiological shear. We tested both arterial (1,000 sec^−1^) and venous shear (200 sec^−1^) [30]. At 1,000 sec^−1^, the tensile strength of the binding of platelets to A*β* peptides is not sufficient to guarantee effective adhesion and thrombus formation (Figures 5(a) and 5(b)). At lower shear rate corresponding to venous circulation (200 sec^−1^), A*β*1-42 but not A*β*1-40, A*β*25-35, or scrambled A*β*1-42 induced convincing platelet adhesion and thrombus formation (Figures 5(c), 5(e) and 5(f)). Similarly to what was observed in static conditions, the inhibition of NOX with VAS2870 (10 *μ*M) inhibited platelet adhesion (and thrombus formation) in response to A*β*1-42 (Figures 5(d) and 5(g)).

**Figure 6:**
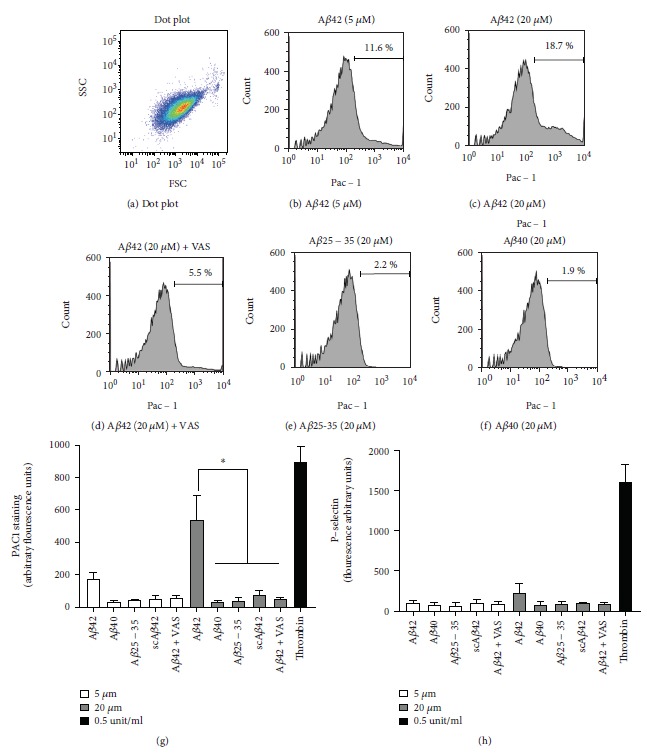
Activation of integrin *α*IIb*β*3 by A*β*1-42. Washed platelets were stimulated as indicated with A*β*1-42, A*β*1-40, A*β*25-35, or scrambled A*β*1-42 or 0.5 units/ml thrombin for 10 minutes and then labelled with FITC-PAC1 (b-g) and PE-Cy5-P-selectin (h) for a further 10 minutes. A side-scattering (SSC)/forward-scattering (FSC) dot plot is shown in (a) and suggests the high purity of the platelet preparation. The histograms for the intensity of PAC1 staining in the different conditions are shown in (b-f) (representative of 3 independent experiments). Data analyses are shown in (g) and (h). Statistical analysis by one-way ANOVA with Bonferroni posttest is shown in (g) and (h) (*n* = 3, ^∗^*P* < 0.05).

As the mechanisms leading to platelet adhesion to A*β*1-42 remain unclear, but previous work suggest a role for integrin *α*IIb*β*3 on the adhesion to A*β*1-40 [22, 31], we tested the activation of this integrin using the antibody PAC1 by flow cytometry. These experiments suggested that only A*β*1-42 (but not A*β*1-40, A*β*2535, or scrambled A*β*1-42) induced a convincing activation of integrin *α*IIb*β*3 (Figures 6(a)–6(g)). NOX inhibition with 10 *μ*M VAS2870 abolished A*β*1-42-induced *α*IIb*β*3 activation (Figure 6(d)). Interestingly, no significant translocation of P-selectin to the surface of the platelets was observed, suggesting that A*β* peptides (including A*β*1-42) cannot stimulate effective platelet degranulation on their own.

**Figure 7:**
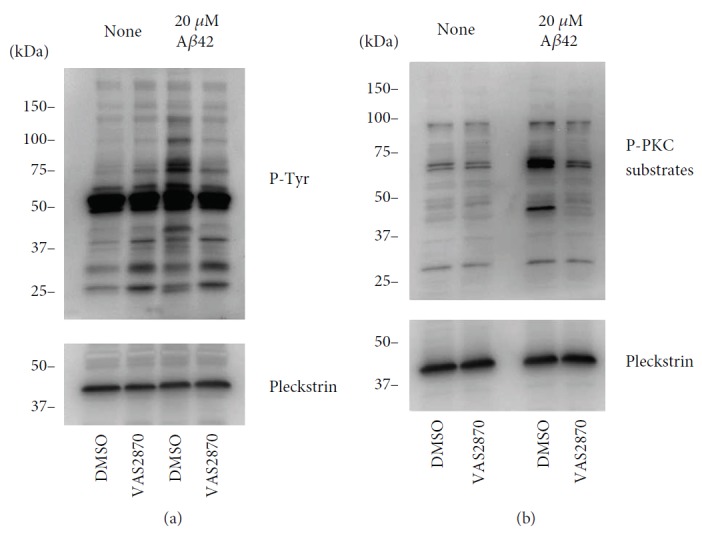
A*β*42-induced signalling in platelets. (a, b) Unstimulated and A*β*42-stimulated (20 *μ*M) human platelets were treated with 10 *μ*M DMSO or VAS2870. Total proteins were separated by SDS-PAGE as described in the Materials and Methods, and protein phosphorylation was analysed by immunoblotting with the indicated antibodies: (a) anti-p-Tyr and anti-pleckstrin antibody and (b) anti-PKC phosphosubstrate and anti-pleckstrin antibody. The figure represents blots from three independent experiments.

We next investigated the effect of A*β*1-42 on intracellular platelet signalling using phosphospecific immunoblotting. Both unstimulated and A*β*1-42-stimulated human platelets were treated with either DMSO (control) or VAS2870. They were then lysed, and the resulting protein extracts were separated by SDS-PAGE. Immunoblotting for phosphotyrosine and phosphorylated PKC substrates is shown in Figure 7. These antibodies are used to determine whether tyrosine phosphorylation cascades and PKC are activated by A*β*1-42 treatment, but they do not allow the identification of the targets of the phosphorylation events and function as a qualitative evidence of activation of the abovementioned signalling pathways. In these experiments, pleckstrin is used simply as a loading control. Tyrosine phosphorylation is one of the key events that occur upon platelet activation, therefore detecting tyrosine phosphorylation of platelet proteins provides a proof that A*β*1-42 induces platelet signalling activation.  Figure 7(a) shows the phosphotyrosine profile of resting and A*β*1-42-treated platelets in the absence or presence of VAS2870. Several bands are observed upon stimulation with A*β*1-42 (compared to DMSO-treated controls), which suggests activation of tyrosine kinase-dependent pathways and generation of tyrosine-phosphorylated protein substrates. Very significantly, the pretreatment with VAS2870 leads to the abolishment of tyrosine phosphorylation in response to A*β*1-42, which suggests that the activity of NADPH oxidases is necessary for the signalling induced by this peptide.

PKC is an essential protein kinase enzyme that is activated by diacylglycerol (DAG) and Ca^2+^ released from internal stores. The activation of PKC is a well-known intracellular event induced by platelet activation, which is usually accompanied by phosphorylation of regulatory serine/threonine residues in PKC substrate proteins [32–34]. In Figure 7(b), several bands corresponding to different substrate proteins for PKC appear more intensely upon A*β*1-42 stimulation, suggesting that PKC is activated by this peptide. PKC activation is strongly associated to platelet activation and induction of thrombus formation [34–36]. The abolishment of phospho-PKC immunostaining by VAS2870 suggests that NADPH oxidase activity is required for the A*β*1-42-dependent platelet signalling leading to PKC activation.

## 4. Discussion

The differential ability of the tested *β* amyloid peptides to induce platelet adhesive responses in static and flow conditions is extremely interesting, but it remains difficult to explain. Nonetheless, our observations are not isolated, as A*β*1-42 has been identified as the most biologically active of the amyloid peptides [37–39]. The biological activity of A*β*1-42 is associated to a marked toxicity of this peptide in several experimental systems [37–39]. One possible explanation is the marked propensity of A*β*1-42 to form fibrils compared to A*β*1-40 [40], which is due to the promotion of intermolecular interactions between amyloid monomers induced by the hydrophobic properties of the extra amino acids of A*β*1-42 compared to A*β*1-40. Recent studies have confirmed the enhanced propensity of A*β*1-42 to form fibrils compared to other A*β* peptides [41, 42]. This draws an interesting parallel with the effect of collagen on platelets. The activation of GPVI on platelets and induction of thrombus formation depends on the fibrillary structure of collagen (i.e., monomeric collagen does not induce platelet activation) [43]. Therefore, similarly to collagen, the *β* amyloid peptides may also need to be in a fibrillar form to bind adjacent receptors and induce effective intracellular signalling in platelets. This seems to favour the hypothesis that GPVI is the receptor for *β* amyloid peptides on platelets, as this receptor preferentially binds substrates in a fibrillary form, which allows the contemporaneous interaction of the same fibril to different receptors (GPVI and integrin *α*2*β*1 in the case of collagen) [44, 45].

Our static adhesion results are in partial disagreement with older studies describing the ability of A*β*25-35 and A*β*1-40 to promote platelet adhesion [16, 22, 31, 46]. In reality, in our experiments, A*β*1-40 and A*β*25-35 coating led to a noticeable increase in platelet adhesion (from less than 200 platelets per optical field on scrambled peptides to around 400). Possibly because of the extent of the effect of A*β*1-42 (over 800 platelets per optical field), the effect of these two peptides did not have statistically significant results. Analysis of adhering platelets at a higher magnification revealed that A*β*1-40 and A*β*25-35 did not induce extensive platelet spreading, with most platelets adhering to these substrates displaying a spherical morphology and modest filopodia formation, which is indicative of partial activation. These data are therefore suggesting some ability of A*β*25-35 and A*β*1-40 to induce platelet adhesion, but a significantly higher platelet adhesion to A*β*1-42, which is likely to induce more extensive platelet intracellular signalling and full spreading (as suggested by spreading data on Figure 3). The conditions utilised for the resuspension of the peptides and the coating of the surfaces in this and Canobbio et al.'s study of 2013 [16] are different, which is likely to affect the level of peptide fibrillation and ability to bind platelets. Further investigation of this discrepancy is necessary to fully understand how platelet binding of *β* amyloid peptides is regulated. As platelet adhesion under static conditions recapitulates platelet adhesion in the bloodstream, these data suggest the possibility that microthrombosis observed in the neurovasculature of AD patients is due to platelet adhesion to A*β*1-42 accumulating in the perivascular space and migrating into the bloodstream via endothelial cell transport [47].

We also tested A*β* peptides for their ability to induce thrombus formation in whole blood under physiological shear. In previous studies, it was shown that A*β*25-35 was not able to induce thrombus formation on its own [35]. This was confirmed in the present study. The ability of amyloid peptides to potentiate platelet adhesion on collagen that we showed in previous studies was not investigated in this manuscript because the A*β*1-42 peptide showed a remarkable ability to induce platelet adhesion on its own. Although A*β*1-42 has been shown to potentiate platelet adhesion to collagen and other substrates previously [14, 48], here we present the first evidence that this peptide alone is sufficient to induce thrombus formation under flow.

Integrin *α*IIb*β*3 has been suggested as the receptor on platelets for A*β*1-40 [22, 31]. Therefore, we analysed whether this integrin is activated in the presence of A*β* peptides. Integrins are adhesion receptors characterised by two activation states (active and inactive), with only the active state able to interact and bind its substrates. The signalling leading to integrin activation is known as inside-out signalling, while the signalling triggered by the engagement of the integrin with its substrate is known as outside-in signalling [49]. With the PAC1 antibody, we were able to assess the activation of *α*IIb*β*3, which is significant for A*β*1-42 (but not for A*β*1-40, A*β*25-35, or scrambled A*β*1-42). This is a significant finding suggesting profound differences in the biological effect of A*β* peptides, with only A*β*1-42 inducing signalling activation in platelets. This is in contrast with previous studies showing the signalling response induced by A*β*1-40 [22, 31] or A*β*25-35 [35]. This discrepancy remains difficult to explain, but the differences in the experimental conditions and the preparation of the peptide are a likely explanation. In addition, our current study cannot categorically exclude some level of platelet activation by other A*β* peptides (as shown, e.g., in the static adhesion experiments where A*β*1-40 and A*β*25-35 induce a moderate increase compared to controls). Certainly, A*β*1-42 represents by far the most active A*β* peptide in our hands.

One important question that remains unanswered relates to the receptor responsible for the initial engagement of A*β*1-42. Integrin *α*IIb*β*3 is the most expressed and functionally crucial adhesion receptor in platelets [50]. Its activation is the consequence of a signalling cascade known as inside-out signalling, which requires receptor-dependent activation. Therefore, although integrin *α*IIb*β*3 is likely to participate in platelet adhesion to A*β* peptides, an alternative receptor is likely to exist. Different receptors have been suggested, including protease-activated receptor 1 [23], GPVI [27], and CD36 [21]. Our current data do not help to resolve this impasse.

The intracellular signalling involved in platelet adhesion and activation by *β* amyloid peptides has been studied by several groups. For example, Sonkar et al. showed that exposure to A*β*25-35 resulted in increased myosin light chain (MLC) phosphorylation and RhoA-GTP levels. This led to the conclusion that A*β*25-35 induces cellular activation via RhoA-dependent modulation of actin and cytoskeletal reorganisation [51]. Our previous investigations also showed that A*β*25-35 promoted intracellular calcium increase by entry from the extracellular environment, which led to dense granule and ADP release, and in turn to the activation of the P2Y_12_ receptor, the small GTPase Rap1b, and both PI3K and MAP kinase pathways [35]. In this study, we utilised tyrosine phosphorylation and PKC-dependent phosphorylation tested by immunoblotting (Figures 7(a) and 7(b)) as markers of platelet activation and to confirm that NOX activity is crucial to trigger A*β*-dependent signalling in platelets. No further detail on the signalling cascades triggered by A*β* peptides can be drawn from this study. The activation of tyrosine phosphorylation and PKC-dependent protein phosphorylation cascades are central to platelet activation and common to most platelet agonists [52, 53]. We have shown previously the modulation of PKC activity by NOX inhibitors, possibly via dampening of GPVI receptor signalling [25]. Other investigators highlighted the link between NOX activation and PKC activity. In fact, this appears to be a bidirectional interaction, not only with different PKC isozymes showing the ability to activate NOXs (e.g., [54]) but also with NOX-dependent ROS leading to oxidation and activation of PKC enzymes [55]. The data from our current study could be explained by either direct PKC stimulation or triggering of cell signalling leading to PKC activation.

Interestingly, although the effect on *α*IIb*β*3 by A*β*1-42 was very evident (i.e., similar to thrombin for 20 *μ*M A*β*1-42), there was no apparent platelet degranulation, as measured by P-selectin immunostaining. This implies that differently to canonical agonists such as thrombin, collagen, or thromboxane A2, A*β*1-42 induces integrin activation without full platelet activation (i.e., partial stimulation). This may explain the poor activity of A*β*1-42 as a platelet agonist in some traditional assays, such as platelet aggregation [28].

In general, the variability in the peptides utilised (e.g., A*β*25-35, A*β*1-40, or A*β*1-42) and the focus of different studies on different receptors and signalling pathways led to apparently contradicting results. For example, an intriguing study reported the reduction of A*β* peptide-dependent platelet activation by fibrinogen [56]. Although the underlying mechanisms remain difficult to explain, this observation may be correlated to our data on A*β* peptide-dependent activation of integrin *α*IIb*β*3 (which is the main fibrinogen receptor on platelets). The use by authors of the above study of a different A*β* peptide for their stimulations (i.e., A*β*25-35) makes any comparison of our and their studies difficult. Further studies are required to resolve these contradictions.

This study highlights the importance of NADPH oxidase activation and platelet oxidative responses in the prothrombotic responses induced by A*β*1-42, which is the *β* amyloid peptide accumulating in the brain of Alzheimer's and cerebral amyloid angiopathy (CAA) patients. In addition to giving us some direction in the elucidation of the molecular mechanisms underlying platelet activation by *β* amyloid peptides, these data suggest a potential therapeutic opportunity aiming at limiting the vascular component of Alzheimer's disease by targeting NADPH oxidase activity.

## Data Availability

The manuscript does not contain data-intensive results and did not require the use of online repositories. Raw data are available on request by contacting the corresponding author.
